# Dataset on aerosol loading and deposition over Nouakchott-Mauritania

**DOI:** 10.1016/j.dib.2018.07.018

**Published:** 2018-07-09

**Authors:** M.E. Emetere, G.A. Adeyemi

**Affiliations:** a Department of Physics, Covenant University Canaan Land, P.M.B 1023, Ota, Nigeria; b Department of Civil Engineering, Covenant University Canaan Land, P.M.B 1023, Ota, Nigeria; c Department of Mechanical Engineering and Science, University of Johannesburg, APK, South Africa

**Keywords:** Air pollution, Aerosol, Nouakchott, Mauritania, Model

## Abstract

Aerosol optical depth (AOD) is a vital parameter that determines air quality over a geographic enclave. In this paper, the pollution state of Nouakchott-Mauritania was considered. Fifteen years primary (aerosol optical depth) dataset was obtained from the Multi-angle Imaging Spectro-Radiometer (MISR). The secondary datasets were generated from the primary dataset to understand the short and long term effect of aerosol loading over nouakchott. The dataset is important to resolve the ground effect of satellite measurements.

**Specifications Table**

**Table:** 

Subject area	Air Pollution
More specific subject area	Aerosol loading and Retention
Type of data	Tableand figure
How data was acquired	Multi-angle Imaging Spectro-Radiometer (MISR).
Data format	Raw and analyzed
Experimental factors	Aerosol Optical Depth
Experimental features	Measurement at 550 nm
Data source location	Nouakchott-Mauritania
Data accessibility	Multi-angle Imaging Spectro-Radiometer

**Value of the data**

• The data gives a good background for further study on aerosol loading.
• The data provides technician necessary insight towards configurating sun-photometer over Nouakchott-Mauritania.
• The data helps to quantify the extent of air pollution.
• The data provides modeller necessary insight on aerosol loading and retention challenges over Nouakchott-Mauritania.

## 1 Data

One of the known methods for examining the level of pollution over an area is the aerosol optical depth (AOD). Optical properties of aerosol particles have severe influence over the local radiative forcing and radiation balance of the earth [1], [2]. The interaction between aerosol and solar radiation can be described by its optical properties. The optical parameters used to describe the aerosol-solar radiation are the extinction and scattering coefficients, the aerosol depth and the single-scattering phase [3], [4], [5]. From the AOD dataset, aerosol hygroscopic growth factor, total atmospheric optical thickness and aerosols loading [6], [7].

The primary data was obtained from Multi-angle Imaging Spectro-Radiometer (MISR) i.e. found in Table 1A, Table 1B, Table 1C. The tunning and atmospheric constants for fifteen was obtained using the West African regional scale dispersion model (WASDM) from the AOD dataset (Fig. 2, Fig. 3). The tunning and atmospheric constants are factors that determines the accuracy of ground instruments e.g. sun photometer [6], [7] and they are presented in Table 2. The secondary dataset i.e. aerosol loading was generated using the extended WASDM are presented in Table 3A-C.

**Table 1A:** Summarized aerosol optical depth dataset over Nouakchott.

**Month**	**2000**	**2001**	**2002**	**2003**	**2004**	**2005**
**Jan**	0.8695	0.276	0.3025	0.233833333	0.1994	0.3684
**Feb**	0.9052	0.36775	0.48725	0.4885	0.384	0.392666667
**Mar**	0.896	0.4088	0.5454	0.4325	0.592166667	0.2902
**Apr**	0.70325	0.7436	0.337	0.5725	0.552	0.6452
**May**	0.9278	0.31325	0.528666667	0.831666667	0.43375	0.746333333
**Jun**	0.9865	0.7084	0.8676	0.8752	0.7165	0.610833333
**Jul**	0.781166667	0.7262	0.90075	0.7845	0.971333333	0.9316
**Aug**	0.4696	0.8254	0.7206	0.905	0.655	0.91825
**Sep**	0.2405	0.9795	0.753333333	0.7454	0.6658	0.725666667
**Oct**	0.3702	0.5575	0.6165	0.5506	0.445666667	0.4772
**Nov**		0.4895	0.4306	0.2888	0.37525	0.3386
**Dec**		0.21075	0.2322	0.3652	0.301833333	0.4032

**Table 1B:** Summarized aerosol optical depth dataset over Nouakchott.

**Month**	**2006**	**2007**	**2008**	**2009**	**2010**
**Jan**	0.251	0.49375	0.463	0.2694	0.273333333
**Feb**	0.351333333	0.418	0.5065	0.337	0.2435
**Mar**	0.4844	0.3786	0.5715	0.5065	0.6442
**Apr**	0.770666667	0.3885	0.9355	0.473833333	0.6066
**May**	0.6445	0.680833333	0.514666667	0.7228	0.764
**Jun**	0.8135	0.701333333	0.6185	0.689333333	0.673166667
**Jul**	0.915833333	0.8174	0.96	0.793666667	0.947666667
**Aug**	0.8032	0.901333333	0.76425	0.783	0.802833333
**Sep**	0.6875	0.76175	0.665333333	1.003	0.6295
**Oct**	0.591666667	0.5162	0.603	0.374166667	0.36275
**Nov**	0.2605	0.3746	0.3645	0.25075	0.22325
**Dec**	0.48975	0.328166667	0.34325	0.143333333	0.262333333

**Table 1C:** Summarized aerosol optical depth dataset over Nouakchott.

**Month**	**2011**	**2012**	**2013**
**Jan**	0.2624	0.3988	0.5962
**Feb**	0.243	0.28	0.2964
**Mar**	0.2772	0.756666667	0.2044
**Apr**	0.647333333	0.649666667	0.4878
**May**	0.471	0.742333333	0.484
**Jun**	0.588666667	0.871666667	0.700666667
**Jul**	0.7044	0.762666667	0.731
**Aug**	0.6412	0.600833333	0.597166667
**Sep**	0.572	0.533833333	0.4185
**Oct**	0.546333333	0.25	0.3666
**Nov**	0.284666667	0.25875	0.175
**Dec**	0.308	0.188

**Table 2:** Atmospheric constants over Nouakchott.

**Location**	$a_{1}$	$a_{2}$	$n_{1}$	$n_{2}$	α	**Β**
Nouakchott	0.9442	0.8131	0.4369	0.08213	$\pm \frac{\pi }{8}$	$\pm \frac{\pi }{8}$

## 2 Experimental design, materials and methods

**Fig. 1:**
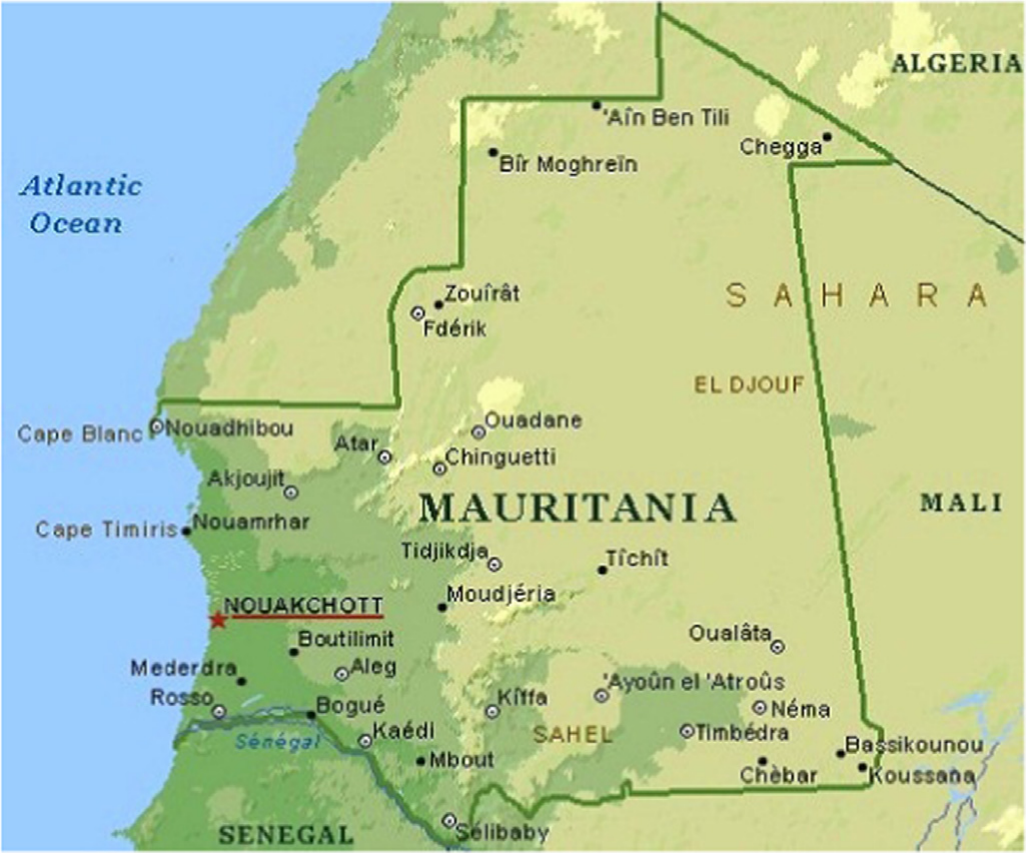
Geographical map of Nouakchott.

Mauritania is located on latitude 16°N to 22°N and longitude 7°W to 17°W. It is bounded within an approximate total area of 1,030,700 km^2^. Its geographical structure includes arid plains, cliff, plateau and oases. Its climate is hot with irregular rainfall. Nouakchott is located on longitude and latitude of 18.09° and −15.98° (Fig. 1).

The West African regional scale dispersion model (WASDM) for calculating aerosol loading over a region:

(1) $$\psi \left(\lambda \right)={a_{1}}^{2}\cos\left(\frac{n_{1}\pi \tau (\lambda )}{2}x\right)\cos\left(\frac{n_{1}\pi \tau (\lambda )}{2}y\right)+\ldots \ldots {a_{n}}^{2}\cos\left(\frac{n_{n}\pi \tau (\lambda )}{2}x\right)\cos\left(\frac{n_{n}\pi \tau (\lambda )}{2}y\right)$$

a is atmospheric constant gotten from the fifteen years aerosol optical depth (AOD) dataset from MISR, *n* is the tunning constant, τ(λ) is the AOD of the area and ψ(λ) is the aerosol loading. The analysis of Eq. (1) was done using the C++ codes.

**Fig. 2:**
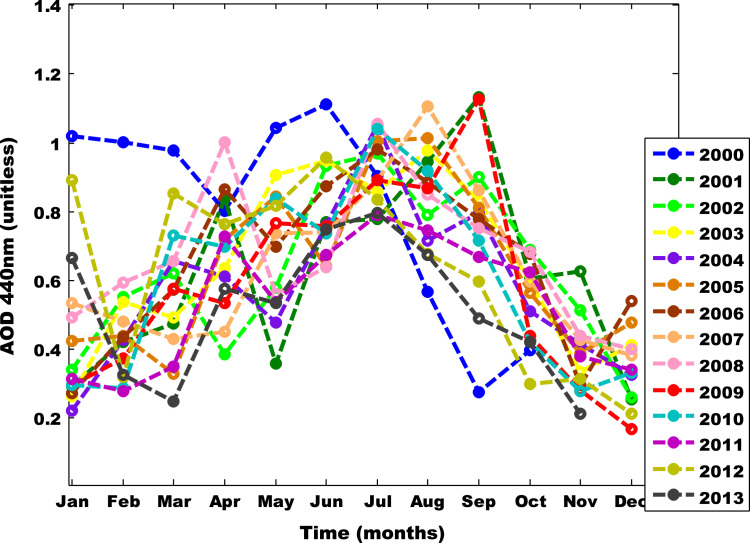
AOD pattern for Nouakchott 2000 – 2013.

The value of the atmospheric and tuning constant for fifteen years was determine using Eq. (1) over fifteen years data (Fig. 1, Fig. 2). The summary of the AOD is shown in Table 1. The value atmospheric and tuning constant i.e. obtained from the comprehensive dataset is shown in Table 2 . The secondary dataset i.e. aerosol loading was generated using the extended WASDM are presented in Table 3A, Table 3B, Table 3C. The percentage of the highest aerosol loading is shown in Table 4.

**Fig. 3:**
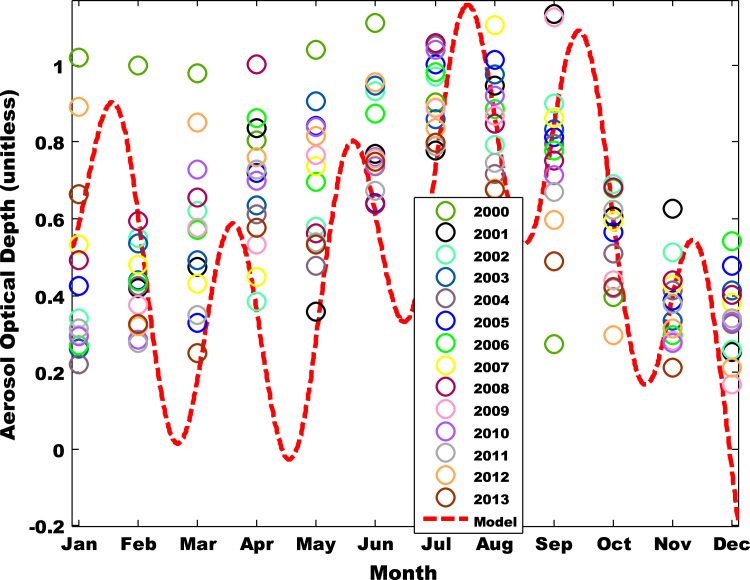
AOD for new model and MISR ( Nouakchott, 2000-2013).

**Table 3A:** Aerosol loading over Nouakchott.

**Month**	**2000**	**2001**	**2002**	**2003**	**2004**
**Jan**	0.610251849	0.859940716	0.853678054	0.868774357	0.874939468
**Feb**	0.589772356	0.83597879	0.795581598	0.795107337	0.831078864
**Mar**	0.595085002	0.823232557	0.77245375	0.815325887	0.752338345
**Apr**	0.699664467	0.679005349	0.844718376	0.760956028	0.76969456
**May**	0.576626083	0.850983355	0.779328727	0.631523544	0.814897949
**Jun**	0.54192479	0.697069678	0.611331169	0.607007215	0.692963222
**Jul**	0.659127654	0.688005495	0.592344977	0.65733573	0.550959607
**Aug**	0.802167699	0.635000181	0.690872978	0.589888095	0.723341959
**Sep**	0.867471212	0.546100116	0.673912489	0.678066626	0.718144143
**Oct**	0.835252388	0.767374939	0.741367625	0.770282069	0.810763945
**Nov**	0.89151364	0.794727189	0.815974272	0.856983577	0.833741237
**Dec**	0.89151364	0.87301247	0.869088235	0.83673016	0.853842252

**Table 3B:** Aerosol loading over Nouakchott.

**Month**	**2005**	**2006**	**2007**	**2008**	**2009**
**Jan**	0.835786501	0.865347273	0.793104241	0.804577017	0.861415644
**Feb**	0.828387115	0.84073205	0.820210232	0.788164773	0.844718376
**Mar**	0.856652431	0.796659063	0.832728512	0.761388138	0.788164773
**Apr**	0.728005794	0.664742877	0.829688	0.572117676	0.800606934
**May**	0.677579322	0.728336983	0.710809619	0.784945797	0.689748183
**Jun**	0.743952326	0.641563385	0.700626936	0.740451104	0.706612513
**Jul**	0.574402948	0.583603489	0.639418083	0.557681382	0.652385237
**Aug**	0.582197336	0.647202074	0.592008051	0.668152109	0.658142646
**Sep**	0.688279207	0.707520791	0.66947573	0.718369984	0.532048552
**Oct**	0.79935715	0.75256023	0.784336658	0.74749559	0.834066983
**Nov**	0.844281011	0.863350584	0.833936788	0.836935581	0.865398856
**Dec**	0.825042825	0.794632049	0.847098337	0.842999029	0.882923782

**Table 3C:** Aerosol loading over Nouakchott.

**Month**	**2010**	**2011**	**2012**	**2013**
**Jan**	0.860540741	0.862942723	0.826449479	0.750543205
**Feb**	0.866873282	0.866973432	0.859030287	0.855167722
**Mar**	0.728478842	0.859668896	0.672158962	0.874103366
**Apr**	0.745871499	0.726994853	0.725886377	0.79537305
**May**	0.668284589	0.801652868	0.679665079	0.796809857
**Jun**	0.714564347	0.753888506	0.609019683	0.700961295
**Jul**	0.56496546	0.699086143	0.668990707	0.685536266
**Aug**	0.647402069	0.729894787	0.748469507	0.750111577
**Sep**	0.735370769	0.761172157	0.777224566	0.820044262
**Oct**	0.837447557	0.772065182	0.865553311	0.836318235
**Nov**	0.870770385	0.857952393	0.863723734	0.878729234
**Dec**	0.862957082	0.852310418	0.876770215	0.89151364

**Table 4:** Percentage of increase of aerosols loading over Nouakchott.

**Year**	2001	2004	2007	2009
Percentage (%)	29.1	3.6	4.7	8.0
